# Supplementary figures and images for: Factors influencing the distribution of woody plants in tropical karst hills, south China
Source: PeerJ. 2023 Oct 27;11:e16331. doi: 10.7717/peerj.16331 (PMC10615033; doi:10.7717/peerj.16331)

# Bray-Curtis Anosim

$R = 0.588$  ,  $P = 0.001$

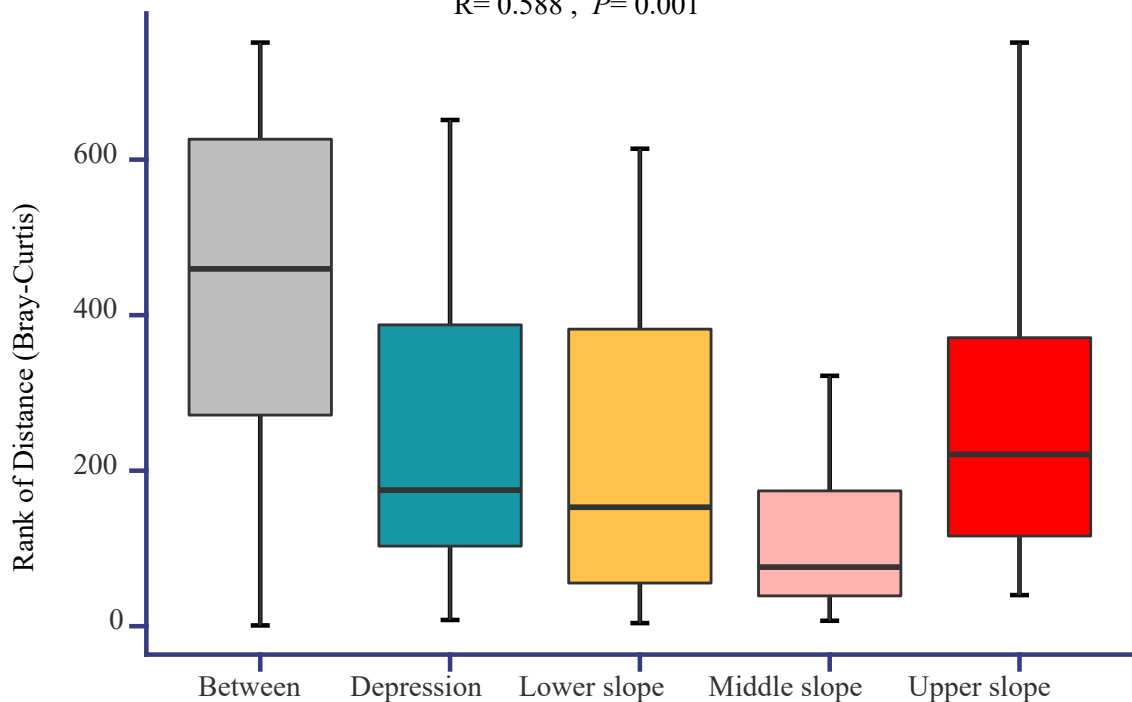

Supplement: Supplemental Information 1 [file peerj-11-16331-s001.pdf]
