# Supplementary material for: Factors influencing the distribution of woody plants in tropical karst hills, south China
Source: PeerJ. 2023 Oct 27;11:e16331. doi: 10.7717/peerj.16331 (PMC10615033; doi:10.7717/peerj.16331)
Supplement: Supplemental Information 2 [file peerj-11-16331-s002.docx]

| **Plot No.** | **Latitude** | **Longitude** | **Slope degree (°)** | **Rock outcrop rate (%)** | **Elevation(m)** | **Slope aspect (°)** |
| --- | --- | --- | --- | --- | --- | --- |
| DE1 | 22°31′50.25″ | 106°48′51.73″ | 6 | 30 | 205 | 92 |
| DE2 | 22°31′49.40″ | 106°48′52.39″ | 11 | 30 | 112 | 270 |
| DE3 | 22°28′33.65″ | 106°54′54.70″ | 3 | 35 | 216 | 27 |
| DE4 | 22°27′40.22″ | 106°54′13.57″ | 0 | 5 | 158 | 12 |
| DE5 | 22°27′28.40″ | 106°54′28.82″ | 0 | 5 | 226 | 205 |
| DE6 | 22°28′02.59″ | 106°53′50.47″ | 11 | 40 | 198 | 245 |
| DE7 | 22°27′40.82″ | 106°54′09.65″ | 7 | 10 | 204 | 245 |
| DE8 | 22°31′57.68″ | 106°49′31.33″ | 13 | 80 | 201 | 110 |
| DE9 | 22°28′10.87″ | 106°56′27.52″ | 3 | 5 | 206 | 195 |
| DE10 | 22°28′11.73″ | 106°56′20.84″ | 8 | 10 | 200 | 203 |
| LS1 | 22°27′48.78″ | 106°53′59.18″ | 30 | 55 | 240 | 30 |
| LS2 | 22°32′02.53″ | 106°50′19.43″ | 20 | 55 | 296 | 245 |
| LS3 | 22°27′29.24″ | 106°54′20.60″ | 17 | 50 | 210 | 61 |
| LS4 | 22°27′27.52″ | 106°54′30.36″ | 35 | 45 | 224 | 332 |
| LS5 | 22°32′01.41″ | 106°49′38.64″ | 25 | 80 | 278 | 27 |
| LS6 | 22°27′26.82″ | 106°56′53.81″ | 40 | 65 | 253 | 329 |
| LS7 | 22°27′17.99″ | 106°56′59.71″ | 35 | 55 | 310 | 235 |
| LS8 | 22°27′15.23″ | 106°57′06.06″ | 25 | 80 | 315 | 183 |
| LS9 | 22°32′07.67″ | 106°50′14.18″ | 20 | 50 | 289 | 216 |
| LS10 | 22°31′56.53″ | 106°49′29.69″ | 27 | 75 | 302 | 128 |
| MS1 | 22°31′47.59″ | 106°48′54.03″ | 27 | 45 | 340 | 264 |
| MS2 | 22°32′00.84″ | 106°50′21.13″ | 30 | 60 | 338 | 245 |
| MS3 | 22°28′28.17″ | 106°54′48.40″ | 40 | 60 | 269 | 239 |
| MS4 | 22°28′31.33″ | 106°54′51.06″ | 35 | 65 | 265 | 22 |
| MS5 | 22°28′32.97″ | 106°54′50.43″ | 40 | 55 | 292 | 30 |
| MS6 | 22°27′41.20″ | 106°57′17.35″ | 30 | 65 | 325 | 33 |
| MS7 | 22°27′43.89″ | 106°57′11.25″ | 25 | 65 | 301 | 218 |
| MS8 | 22°27′24.57″ | 106°54′19.18″ | 30 | 65 | 277 | 130 |
| MS9 | 22°27′15.34″ | 106°57′00.90″ | 30 | 70 | 315 | 285 |
| MS10 | 22°27′13.15″ | 106°57′04.08″ | 40 | 65 | 295 | 7 |
| US1 | 22°28′36.27″ | 106°54′42.71″ | 32 | 70 | 378 | 213 |
| US2 | 22°28′37.75″ | 106°54′57.00″ | 37 | 68 | 335 | 290 |
| US3 | 22°27′34.23″ | 106°57′21.03″ | 30 | 65 | 390 | 242 |
| US4 | 22°27′29.78″ | 106°57′25.23″ | 33 | 55 | 454 | 281 |
| US5 | 22°27′18.38″ | 106°58′10.03″ | 24 | 45 | 461 | 135 |
| US6 | 22°27′16.77″ | 106°58′08.71″ | 29 | 48 | 429 | 125 |
| US7 | 22°27′12.82″ | 106°58′06.29″ | 40 | 90 | 473 | 114 |
| US8 | 22°30′49.21″ | 106°51′47.24″ | 35 | 45 | 470 | 207 |
| US9 | 22°30′48.51″ | 106°51′50.20″ | 33 | 70 | 442 | 194 |
| US10 | 22°28′31.26″ | 106°54′49.17″ | 40 | 65 | 491 | 181 |
