# Supplementary material for: Factors influencing the distribution of woody plants in tropical karst hills, south China
Source: PeerJ. 2023 Oct 27;11:e16331. doi: 10.7717/peerj.16331 (PMC10615033; doi:10.7717/peerj.16331)
Supplement: Supplemental Information 3 — “-”: the family was not ranked in the top 10 or was not found at that slope position. [file peerj-11-16331-s003.docx]

| **Family** | **Depression** | **Lower slope** | **Middle slope** | **Upper slope** |
| --- | --- | --- | --- | --- |
| Euphorbiaceae | 13.457 | 10.874 | 7.648 | 3.941 |
| Moraceae | 13.393 | 18.769 | 6.011 | - |
| Primulaceae | 8.918 | 5.217 | - | - |
| Lauraceae | 7.459 | - | - | 3.652 |
| Rubiaceae | 6.115 | - | 3.631 | 6.652 |
| Phyllanthaceae | 6.056 | 10.081 | 15.697 | 9.319 |
| Malvaceae | 4.960 | 7.122 | 11.926 | 6.837 |
| Araliaceae | 3.316 | - | - | - |
| Burseraceae | 3.255 | 3.028 | - | - |
| Fabaceae | 2.865 | 4.616 | 4.335 | 6.211 |
| Anacardiaceae | - | 7.156 | - | - |
| Annonaceae | - | 4.185 | 5.898 | - |
| Lamiaceae | - | 2.799 | 4.729 | - |
| Achariaceae | - | - | 3.764 | - |
| Putranjivaceae | - | - | 3.380 | - |
| Ebenaceae | - | - | - | 6.775 |
| Sapindaceae | - | - | - | 6.027 |
| Melastomataceae | - | - | - | 4.924 |
| Myrtaceae | - | - | - | 3.790 |
| others | 30.206 | 26.153 | 32.982 | 41.872 |
