# Supplementary material for: Factors influencing the distribution of woody plants in tropical karst hills, south China
Source: PeerJ. 2023 Oct 27;11:e16331. doi: 10.7717/peerj.16331 (PMC10615033; doi:10.7717/peerj.16331)
Supplement: Supplemental Information 4 — “-”: the genus was not ranked in the top 10 or was not found at that slope position. [file peerj-11-16331-s004.docx]

| **Family** | **Genus** | **Depression** | **Lower slope** | **Middle slope** | **Upper slope** |
| --- | --- | --- | --- | --- | --- |
| Moraceae | *Ficus* | 11.531 | 2.262 | 3.183 | - |
|  | *Streblus* | - | 15.767 | - | - |
| Primulaceae | *Ardisia* | 8.120 | 4.833 | - | - |
| Lauraceae | *Litsea* | 4.925 | - | - | - |
| Euphorbiaceae | *Deutzianthus* | 4.453 | 2.574 | - | - |
|  | *Cleidion* | 4.024 | - | - | - |
|  | *Cephalomappa* | - | 6.692 | 3.498 | - |
| Burseraceae | *Garuga* | 2.622 | 2.616 | - | - |
| Rubiaceae | *Metadina* | 2.535 | - | - | - |
|  | *Canthium* | - | - | - | 4.192 |
| Malvaceae | *Sterculia* | 2.441 | 3.497 | 2.267 | - |
|  | *Excentrodendron* | - | - | 5.712 | 3.238 |
| Rhamnaceae | *Ziziphus* | 2.223 | - | - | - |
| Anacardiaceae | *Dracontomelon* | 2.125 | 7.156 | - | - |
| Phyllanthaceae | *Cleistanthus* | - | 7.115 | 14.243 | 5.291 |
| Annonaceae | *Orophea* | - | 2.924 | 3.304 | - |
| Lamiaceae | *Vitex* | - | - | 3.943 | - |
| Achariaceae | *Hydnocarpus* | - | - | 3.764 | - |
| Putranjivaceae | *Drypetes* | - | - | 3.380 | - |
| Violaceae | *Rinorea* | - | - | 3.322 | - |
| Ebenaceae | *Diospyros* | - | - | - | 6.775 |
| Melastomataceae | *Memecylon* | - | - | - | 4.924 |
| Linaceae | *Tirpitzia* | - | - | - | 3.538 |
| Sapindaceae | *Boniodendron* | - | - | - | 3.521 |
| Myrtaceae | *Syzygium* | - | - | - | 2.574 |
| Adoxaceae | *Viburnum* | - | - | - | 2.258 |
| Hypericaceae | *Cratoxylum* | - | - | - | 2.009 |
|  | others | 55.002 | 44.564 | 53.386 | 61.681 |
