# Supplementary material for: Factors influencing the distribution of woody plants in tropical karst hills, south China
Source: PeerJ. 2023 Oct 27;11:e16331. doi: 10.7717/peerj.16331 (PMC10615033; doi:10.7717/peerj.16331)
Supplement: Supplemental Information 5 — “-”: the species was not ranked in the top 10 or was not found at that slope position. [file peerj-11-16331-s005.docx]

| **Family** | **Genus** | **Species** | **Depression** | **Lower slope** | **Middle slope** | **Upper slope** |
| --- | --- | --- | --- | --- | --- | --- |
| Moraceae | *Ficus* | *Ficus hispida* | 9.009 | - | - | - |
|  | *Streblus* | *Streblus tonkinensis* | - | 16.519 | 2.268 | - |
| Euphorbiaceae | *Deutzianthus* | *Deutzianthus tonkinensis* | 4.931 | 3.174 | - | - |
|  | *Cleidion* | *Cleidion bracteosum* | 4.001 | - | - | - |
|  | *Cephalomappa* | *Cephalomappa sinensis* | - | 7.445 | 3.816 | - |
| Lauraceae | *Litsea* | *Litsea variabilis* var. *oblonga* | 4.763 | - | - | - |
| Primulaceae | *Ardisia* | *Ardisia depressa* | 4.441 | - | - | - |
|  |  | *Ardisia thyrsiflora* | 4.135 | 3.176 | - | - |
| Rubiaceae | *Metadina* | *Metadina trichotoma* | 2.512 | - | - | - |
|  | *Canthium* | *Psydrax dicocca* | - | - | - | 4.781 |
| Anacardiaceae | *Dracontomelon* | *Dracontomelon duperreanum* | 2.478 | 7.145 | - | - |
| Burseraceae | *Garuga* | *Garuga pinnata* | 2.474 | - | - | - |
|  |  | *Garuga forrestii* | - | 2.500 | - | - |
| Malvaceae | *Sterculia* | *Sterculia monosperma* | 2.461 | 2.891 | - | - |
|  | *Excentrodendron* | *Excentrodendron tonkinense* | - | - | 5.891 | 3.624 |
|  | *Pterospermum* | *Pterospermum truncatolobatum* | - | - | 2.742 | - |
| Phyllanthaceae | *Cleistanthus* | *Cleistanthus petelotii* | - | 6.901 | - | - |
|  |  | *Cleistanthus sumatranus* | - | - | 14.689 | 3.472 |
| Annonaceae | *Orophea* | *Orophea polycarpa* | - | 3.328 | 3.976 | - |
| Achariaceae | *Hydnocarpus* | *Hydnocarpus hainanensis* | - | 2.500 | 4.318 | - |
| Violaceae | *Rinorea* | *Rinorea bengalensis* | - | - | 3.877 | - |
| Lamiaceae | *Vitex* | *Vitex kwangsiensis* | - | - | 3.708 | - |
| Putranjivaceae | *Drypetes* | *Drypetes perreticulata* | - | - | 3.307 | - |
| Melastomataceae | *Memecylon* | *Memecylon scutellatum* | - | - | - | 5.162 |
| Ebenaceae | *Diospyros* | *Diospyros siderophylla* | - | - | - | 4.124 |
|  |  | *Diospyros eriantha* | - | - | - | 2.545 |
| Sapindaceae | *Boniodendron* | *Boniodendron minus* | - | - | - | 3.907 |
| Linaceae | *Tirpitzia* | *Tirpitzia sinensis* | - | - | - | 3.538 |
| Myrtaceae | *Syzygium* | *Syzygium rehderianum* | - | - | - | 2.858 |
| Adoxaceae | *Viburnum* | *Viburnum triplinerve* | - | - | - | 2.266 |
|  |  | others | 58.794 | 44.421 | 51.409 | 63.722 |
