# Supplementary material for: Factors influencing the distribution of woody plants in tropical karst hills, south China
Source: PeerJ. 2023 Oct 27;11:e16331. doi: 10.7717/peerj.16331 (PMC10615033; doi:10.7717/peerj.16331)
Supplement: Supplemental Information 6 — Note: Different letters of the same row represent significant difference between plant communities among different slope positions (P < 0.05). Values are presented as the means ± standard deviation. [file peerj-11-16331-s006.docx]

| **Environmental variables** | **Depression** | **Lower slope** | **Middle slope** | **Upper slope** |
| --- | --- | --- | --- | --- |
| Slope aspect | 0.63±0.41a | 0.49±0.43a | 0.49±0.43a | 0.77±0.22a |
| Slope degree(°) | 6.20±4.64b | 27.40±7.53a | 32.70±5.64a | 33.30±4.99a |
| Rock outcrop rate(%) | 0.25±0.24b | 0.61±0.13a | 0.62±0.07a | 0.62±0.14a |
| Soil pH | 7.16±0.28a | 7.27±0.27a | 7.42±0.31a | 7.43±0.21a |
| Soil exchangeable calcium(cmol·kg^-1^) | 33.64±16.54c | 47.13±14.75ab | 43.72±9.50bc | 57.25±14.61a |
| Soil organic matter(g·kg^-1^) | 96.88±30.73a | 120.26±35.85a | 115.97±52.99a | 143.74±33.12a |
| Soil total nitrogen(g·kg^-1^) | 5.42±1.63a | 6.33±1.63a | 6.34±0.90a | 6.37±1.66a |
| Soil total phosphorus(g·kg^-1^) | 1.66±0.69a | 1.49±0.57ab | 0.98±0.24bc | 0.66±0.32c |
| Soil total potassium(g·kg^-1^) | 5.62±1.21a | 6.57±2.50a | 3.55±1.46b | 1.82±1.66c |
| Soil water content(%) | 19.86±4.61a | 16.47±4.25ab | 13.91±2.84bc | 12.64±4.30c |
