# Supplementary material for: Factors influencing the distribution of woody plants in tropical karst hills, south China
Source: PeerJ. 2023 Oct 27;11:e16331. doi: 10.7717/peerj.16331 (PMC10615033; doi:10.7717/peerj.16331)
Supplement: Supplemental Information 7 — SR, species richness; SA, slope aspect; SD, slope degree; SP, slope position; ROR, rock outcrop rate; pH, soil pH; SECa, soil exchangeable calcium; SOM, soil organic matter; STN, soil total nitrogen; STP, soil total phosphorus; STK, soil total potassium; SWC, soil water content. *P < 0.05; **P < 0.01. [file peerj-11-16331-s007.docx]

|  | SR | SA | SD | SP | ROR | pH | SECa | SOM | STN | STP | STK |
| --- | --- | --- | --- | --- | --- | --- | --- | --- | --- | --- | --- |
| SA | 0.154 |  |  |  |  |  |  |  |  |  |  |
| SD | 0.085 | -0.113 |  |  |  |  |  |  |  |  |  |
| SP | **0.324^*^** | 0.107 | **0.723^**^** |  |  |  |  |  |  |  |  |
| ROR | -0.231 | -0.124 | **0.565^**^** | **0.499^**^** |  |  |  |  |  |  |  |
| pH | 0.062 | 0.001 | **0.355^*^** | **0.420^**^** | 0.292 |  |  |  |  |  |  |
| SECa | 0.011 | 0.014 | **0.379^*^** | **0.471^**^** | **0.570^**^** | **0.504^**^** |  |  |  |  |  |
| SOM | 0.060 | 0.101 | 0.240 | **0.401^*^** | **0.474^**^** | 0.208 | **0.674^**^** |  |  |  |  |
| STN | -0.157 | -0.040 | 0.213 | 0.219 | **0.556^**^** | 0.186 | **0.700^**^** | **0.666^**^** |  |  |  |
| STP | **-0.478**** | -0.111 | **-0.534**** | **-0.709**** | -0.122 | -0.062 | -0.003 | -0.044 | 0.249 |  |  |
| STK | **-0.471**** | -0.143 | **-0.521**** | **-0.686**** | -0.129 | **-0.368*** | **-0.380*** | **-0.314*** | -0.016 | **0.645^**^** |  |
| SWC | -0.117 | -0.133 | **-0.579**** | **-0.537**** | **-0.485**** | -0.149 | **-0.542**** | **-0.480**** | **-0.446**** | **0.355^*^** | **0.525^**^** |
